# 异基因造血干细胞移植后胰腺萎缩及胰腺外分泌功能不全1例报告及文献复习

**DOI:** 10.3760/cma.j.cn121090-20251203-00566

**Published:** 2026-06

**Authors:** 颖 单, 佩佩 叶, 仁治 裴, 冬 陈, 甜甜 王, 晓薇 史, 滢 陆

**Affiliations:** 宁波大学附属人民医院血液科，宁波 315000 Department of Hematology, the Affiliated People's Hospital of Ningbo University, Ningbo 315000, China

## Abstract

报道1例60岁男性，因“急性B淋巴细胞白血病”接受无关供者外周血异基因造血干细胞移植，回输单个核细胞6.22×10^8^/kg，CD34^＋^细胞6.27×10^6^/kg，移植后第11天血细胞植活，移植后18个月起反复腹泻、体重持续下降，予止泻、抗排异及抗感染治疗无效，经腹部CT、胰腺MRI及粪便胰弹性蛋白酶1检查最终确诊为“胰腺萎缩及重度胰腺外分泌功能不全”，予胰酶替代治疗后症状显著改善。

## Abstract (en)

A 60-year-old male patient with acute B-cell lymphoblastic leukemia received allogeneic hematopoietic stem cell transplantation from an unrelated donor. He was transplanted with 6.22×10^8^ mononuclear cells/kg and 6.27×10^6^ CD34^+^ cells/kg, and engraftment was confirmed at +11 days. Eighteen months post-transplant, the patient developed persistent diarrhea and continued weight loss, which was unresponsive to antidiarrheal, immunosuppressive, and antimicrobial treatments. After abdominal CT, pancreatic magnetic resonance imaging, and fecal elastase-1 tests, he was diagnosed with pancreatic atrophy and severe pancreatic exocrine insufficiency. Symptoms significantly improved following pancreatic enzyme replacement therapy.

异基因造血干细胞移植（allo-HSCT）后部分患者出现难以用移植物抗宿主病（GVHD）[1]或感染[2]解释的腹泻、体重减轻等症状[3]–[4]，与不良预后相关。既往报道表明，胰腺外分泌功能不全（PEI）和胰腺萎缩[5]可能是导致此类患者消化与吸收不良的关键却被忽视的病因[6]–[7]，且胰酶替代治疗（PERT）是首选治疗方法。本文报告1例allo-HSCT后胰腺萎缩及PEI患者并进行文献复习。

## 病例资料

患者，男，60岁，2023年4月30日因“发热1周”入院，确诊“急性淋巴细胞白血病（Ph阴性B细胞，伴ATM、JAK2胚系突变）”，予减低剂量VDP方案（长春地辛、柔红霉素、地塞米松）联合贝林妥欧单抗靶向治疗1个疗程，维奈克拉（VEN）联合贝林妥欧单抗巩固治疗1个疗程，疗效评估完全缓解（CR）。2023年7月17日起予改良Bu/Cy＋ATG方案预处理：阿糖胞苷（Ara-C）4 g·m^−2^·d^−1^, −10～−9 d；白消安3.2 mg·kg^−1^·d^−1^, −8～−6 d；环磷酰胺（CTX）1.8 g·m^−2^·d^−1^, −10～−9 d；抗人T细胞兔免疫球蛋白（ATG）8 mg·kg^−1^·d^−1^, −5～−2 d；司莫司汀250 mg·m^−2^·d^−1^, −3 d。2023年7月27日回输无关供者干细胞：单个核细胞6.22×10^8^/kg，CD34^＋^细胞6.27×10^6^/kg，+11 d血细胞植活。

+54 d发生巨细胞病毒（CMV）血症，给予更昔洛韦、膦甲酸钠抗病毒，CMV特异性免疫球蛋白、利妥昔单抗清除病毒治疗后转阴。移植后3个月停用免疫抑制剂。2024年12月（移植后第17个月）出现皮肤慢性GVHD，予“甲氨蝶呤、甲泼尼龙”抗排异治疗后皮疹好转。期间定期监测骨髓常规示缓解骨髓象，可检测残留病（MRD）持续阴性，短串联重复序列（STR）呈完全嵌合状态。

2025年1月（移植后第18个月）因反复腹泻再次入院，每日解黄色糊状及水样便4～5次，无腹痛，无发热，无便血，予止泻对症治疗后症状无好转，体格检查腹部平软，肠鸣音活跃，无压痛、反跳痛。查外周血CMV DNA阳性（2.22×10^3^拷贝数/ml）；骨髓常规示急性淋巴细胞白血病缓解骨髓象，MRD阴性，STR：99.63％（完全嵌合状态），GVHD因子预测风险低；大便常规+潜血+腺病毒（ADV）阴性，大便碳青霉烯类耐药肠杆菌科细菌检测（CRE）、粪便培养均阴性；腹泻相关病原核酸检测：EB病毒（EBV）、ADV阳性；胸部CT提示肺炎，肠镜未见明显异常。先后予左氧氟沙星联合舒普深、美罗培南抗感染，更昔洛韦、马立巴韦抗病毒，贝舒地尔、甲泼尼龙、环孢素抗排异治疗，生长抑素抑制消化液分泌等对症治疗，肺炎好转，CMV DNA转阴，但腹泻症状无好转，大便性质同前，同时体重进行性下降。由于抗感染、抗排异治疗无效，感染相关腹泻和肠道GVHD诊断不成立。完善腹部CT提示胰腺萎缩（图1A），胰腺MRI提示胰腺实质明显萎缩，胰头部为著（图1B），粪便胰弹性蛋白酶1（FE-1）：53 µg/g（参考值：>200 µg/g提示胰腺外分泌功能正常，100～200 µg/g提示中度胰腺外分泌功能不全，<100 µg/g提示重度胰腺外分泌功能不全），诊断“胰腺萎缩、重度胰腺外分泌功能不全”。予米曲菌胰酶片、胰酶肠溶片PERT，地衣芽孢杆菌活菌胶囊调节肠道菌群，同时环孢素、泼尼松逐渐减量至停药，经积极治疗，患者腹泻明显好转，每日大便1次，基本为成形软便，体重较前增加2.5 kg，目前继续PERT。具体诊疗经过及鉴别诊断思路详见附图1（**扫描本文首页二维码查阅**）。

**图1:**
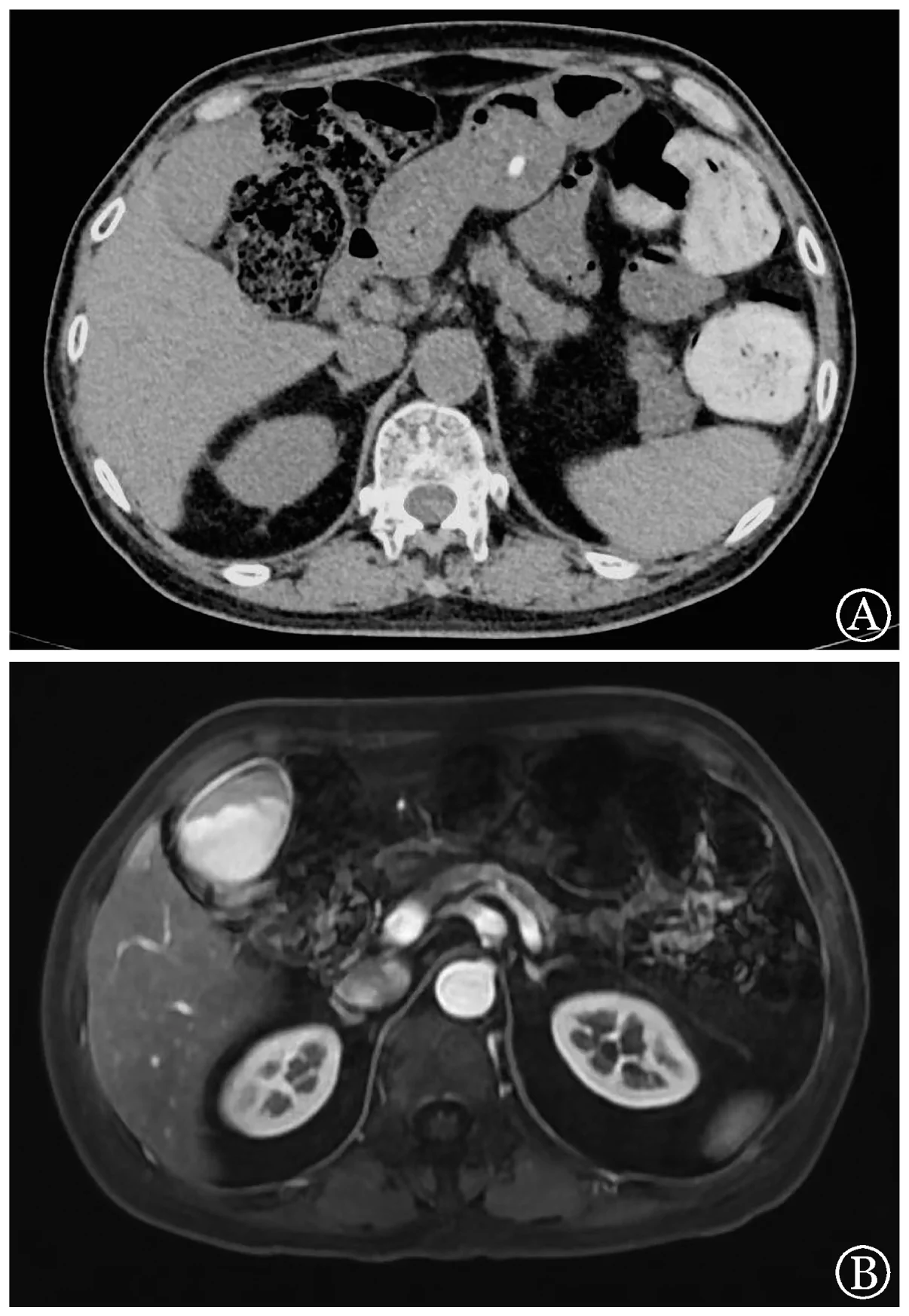
异基因造血干细胞移植后腹部CT（A）、胰腺MRI（B）提示胰腺萎缩

## 讨论及文献复习

PEI定义为因各种原因引起人体自身胰酶分泌不足或分泌不同步，导致患者出现消化吸收障碍[8]–[9]，FE-1检测是国内外广泛报道的非侵入性胰腺外分泌功能检测方法[10]。胰腺萎缩定义为影像学上胰腺外观的体积减少。

早在1991年Jurges等[11]在两个独立病例报道中描述了同种异基因骨髓移植后的继发于胰腺功能不全的腹泻，随后的30多年中，数例HSCT后不明原因腹泻的患者[12]–[13]被先后报道，诊断为严重PEI及胰腺萎缩。而近年来的一些小型临床研究证实，allo-HSCT后PEI及胰腺萎缩并非罕见[14]–[15]。回顾性研究发现胰腺萎缩发生在16.7％～32.4％的allo-HSCT后患者中[5]，前瞻性研究发现有45％的allo-HSCT后患者患有PEI，其中29％的患者表现出持续至少8周的长期PEI迹象[16]。这些数据表明，PEI和胰腺萎缩是allo-HSCT后不可忽视的远期并发症。

更值得关注的是，allo-HSCT后的胰腺萎缩和PEI往往与不良预后相关。研究表明，PEI与更高的继发并发症发生率、更长的肠外营养持续时间、更长的初始住院时间、持续的体重减轻有关,且PEI组的非复发死亡率增加[16]。Okada等[15]的回顾性研究也发现胰腺萎缩与更差的OS显著相关，而胰腺恢复与更好的OS显著相关，强调了在移植后不明原因腹泻患者中对PEI和胰腺萎缩的早期识别和早期干预的重要性。

一、潜在发病机制

allo-HSCT后胰腺萎缩与PEI的发病机制尚未完全阐明，目前认为是免疫[17]与非免疫多重因素综合作用的结果[18]–[19]。结合本例患者的临床特征，我们提出以下机制假设：

1. 预处理毒性：Bu/Cy方案的潜在器官损伤。高剂量化疗预处理是allo-HSCT后器官损伤的重要非免疫机制，本例患者预处理方案中，白消安（Bu）和环磷酰胺（Cy）均为强效烷化剂，可通过DNA链间/链内交联对胰腺细胞产生直接毒性[20]–[21]。动物实验证实Bu/Cy联合预处理亦可导致血管内皮损伤和微循环障碍[22]–[23]，鉴于胰腺对血流灌注高度依赖[24]，可能成为胰腺损伤的机制之一。

2. 免疫介导损伤：GVHD对胰腺外分泌组织的靶向作用。多项研究已证实GVHD与PEI和胰腺萎缩之间存在密切关联。Nakasone等[5]和Okada等[15]发现胰腺萎缩患者发生慢性GVHD（cGVHD）的比例更高，Brook等[14]的回顾性分析也表明与未发生cGVHD患者相比，HSCT后cGVHD患者的平均总胰腺厚度与移植前相比显著下降。Launspach等[16]亦报道PEI组中重度胃肠道GVHD发生率（31％）显著高于对照组（6％）。

GVHD介导胰腺损伤的机制可能涉及以下途径：供者T细胞识别胰腺上皮细胞表面宿主抗原并介导细胞毒性损伤[12]–[13],[25]；cGVHD持续炎症状态可诱导导管周围炎症与纤维化，进而损害胰腺外分泌功能[25]–[26]。此外，Foulis等[27]对26例移植后3个月内死亡患者的胰腺组织学检查证实了GVHD可直接累及胰腺组织，为上述机制提供了病理学依据。

3. 感染触发与加重：病毒感染是免疫受损移植受者胰腺损伤的重要协同因素。CMV可直接侵犯胰腺组织，引起腺泡细胞包涵体形成及局部炎症坏死[28]–[29]；同时通过激活Th1应答、上调促炎细胞因子，理论上可增强供者T细胞对胰腺的免疫攻击[30]。而另一方面，GVHD治疗所需的大剂量免疫抑制剂，通过抑制CMV特异性T细胞功能进一步促进病毒再激活[31]–[32]，形成感染—免疫抑制的恶性循环。Launspach等[16]的前瞻性研究进一步提示ADV感染是HSCT后长期PEI的独立危险因素，而本例患者入院时ADV核酸检测也呈阳性，提示多种因素协同推动胰腺损伤进程。

4. 遗传背景对胰腺损伤易感性的潜在影响：本例患者携带ATM及JAK2胚系突变，可能增加胰腺组织对移植相关损伤的易感性。ATM编码PI3K样蛋白激酶，在DNA双链断裂修复及细胞周期检查点调控中发挥核心作用[33]–[34]。其功能缺陷可降低胰腺细胞对Bu/Cy预处理诱导的DNA损伤修复效率。此外，ATM缺陷可能导致免疫功能异常和促炎细胞因子过度产生[35]–[36]，理论上放大了GVHD及感染介导的免疫和炎症损伤。JAK2/STAT3信号通路在急性胰腺炎中高度激活，JAK2抑制可通过调节细胞因子谱显著改善胰腺炎症[37]，提示JAK2功能异常可能降低胰腺炎症激活阈值，使其在移植相关免疫攻击下出现过度反应，加速胰腺萎缩。

5. 多因素相互作用模型：综合本例的临床特征，我们提出以下“多打击”损伤模型（图2）解释胰腺损伤的发生机制，这一模型亦解释了为何单独抗病毒或抗GVHD治疗效果不佳——胰腺损伤已是多重机制叠加的结果，导致PEI和胰腺萎缩，而非单一病因所致。然而，这些机制假设仍需通过基础研究和临床队列研究加以验证。

**图2:**
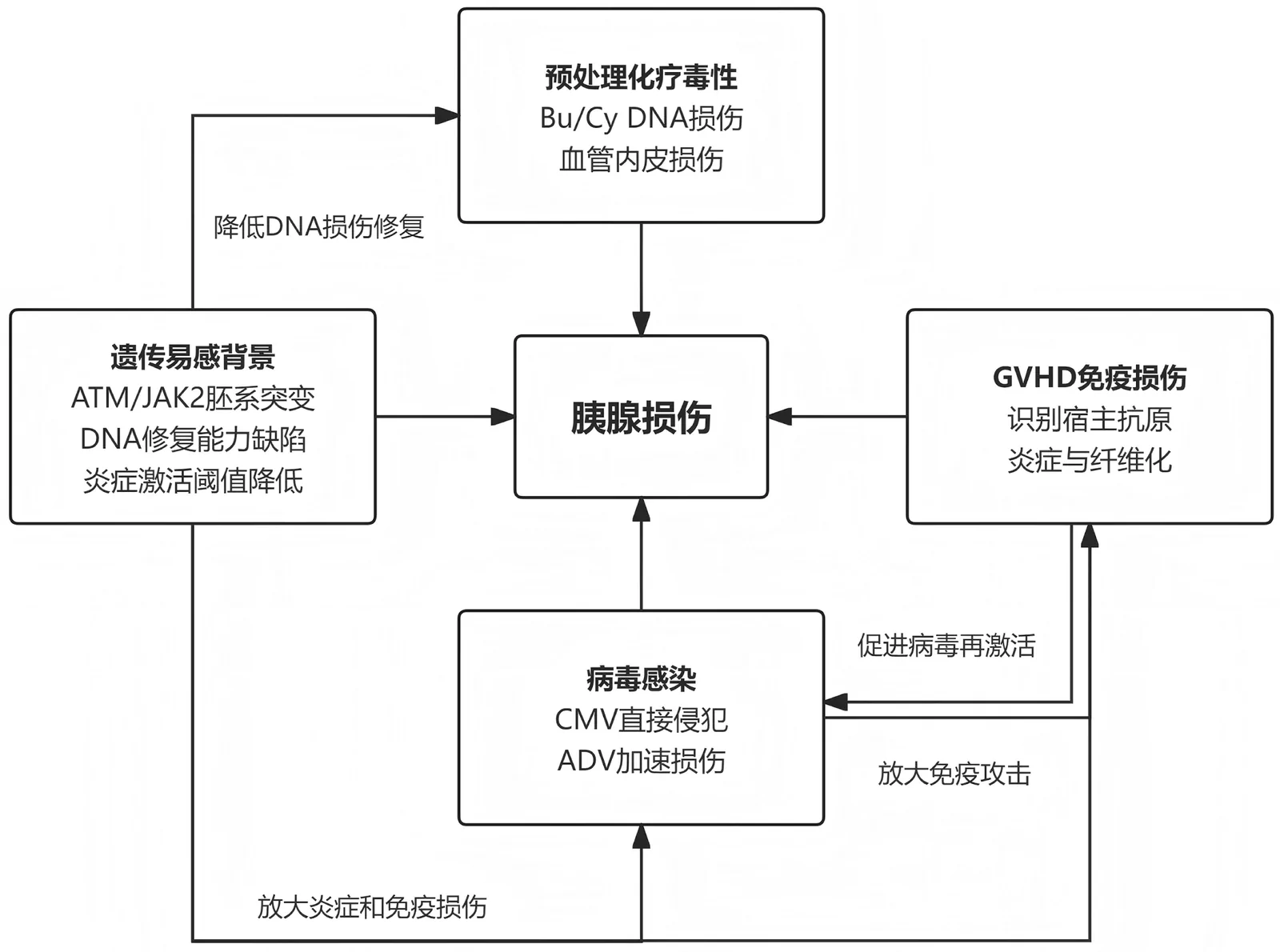
胰腺“多打击”损伤模型图 **注** Bu：白消安；Cy：环磷酰胺；CMV：巨细胞病毒；ADV：腺病毒；GVHD：移植物抗宿主病

二、诊断策略

本例患者allo-HSCT后出现不明原因腹泻，经腹部CT及MRI证实胰腺萎缩、FE-1低至53 µg/g，诊断重度PEI明确，并在PERT后症状显著改善。值得注意的是，患者在确诊前曾同时存在CMV感染及GVHD，两者均可引起腹泻，经积极治疗后CMV DNA转阴、皮疹消退，但腹泻症状持续无改善、体重进行性下降，最终确立PEI为腹泻病因。这一曲折的诊断过程提示在allo-HSCT后腹泻的病因谱中，PEI和胰腺萎缩往往被低估。

基于本例经验与既往文献，建议对allo-HSCT后不明原因腹泻（持续>2周）患者建立以下分层诊断策略：首先排除感染性病因和肠道GVHD；当上述病因排除或经充分治疗后症状仍不改善，特别是伴有体重下降、脂肪泻等临床表现时，应将FE-1检测作为二线诊断手段；对存在GVHD、CMV或ADV感染史、Bu/Cy预处理史等高危因素的患者，可适当提前启动FE-1筛查。腹部CT或者MRI有助于评估胰腺形态学改变，与FE-1互为补充诊断依据。

三、治疗与长期管理

无论何种原因导致的PEI，PERT均是首选治疗方法[38]。据报道，较高的PERT起始剂量对重度PEI患者有效[29]，PERT制剂应与食糜充分混合，抵抗胃液灭活，用营养物从胃排空并快速释放酶[39]，以模拟内源性胰酶的作用。PPI的添加可以提高患者酸性腔内pH值，优化酶释放和提高酶活性[40]。

已经报道的几个病例系列和一个小型队列研究[5]显示胰腺厚度恢复的可能性和PEI的可逆性。但胰腺外分泌的损害并不总是可逆，在HSCT 2年以后，严重的胰腺萎缩和持续的PEI表明永久性胰腺损伤的风险[16]。而本例移植后18个月确诊重度PEI，且伴有胰腺萎缩，提示永久性胰腺损伤的风险较高，患者可能需要终身PERT，应尽早向患者及家属告知长期管理的必要性。

未来需要进一步研究来评估PERT对移植后PEI及胰腺萎缩患者发病率、死亡率的长期影响。

总之，由于allo-HSCT后的胰腺萎缩、PEI往往与不良预后相关，对于移植后出现不明原因的腹泻、体重下降的患者，早期诊断、早期予以PERT带来更好的临床预后。

## Supplementary Material


